# School Students as Drosophila Experimenters

**DOI:** 10.1371/journal.pbio.0030246

**Published:** 2005-07-12

**Authors:** Faiza Siyad, Jodianne Griffiths, Faira Janjua, Elizabeth Jackson, Ian Rodrigues, Fiona Kerr, Daniel Mackay, Simon Lovestone

**Affiliations:** **1**John Ruskin College, Croydon, United Kingdom; **2**Burntwood School, London, United Kingdom; **3**King's College London, London, United Kingdom

## Abstract

Inspired by an undergraduate science project in Drosophila genetics published in *PLoS Biology*, high school students investigate a model of Alzheimer's Disease.

Students can be a valuable resource for the scientific community, as demonstrated by Chen et al. [1]. However, it is not just undergraduate students who can contribute but secondary school students also. Previously some of us have published a Drosophila model of tauopathy where we have shown that overexpression of tau results in disruption of axonal transport and an intact phenotype in both larvae and adults [2]. This phenotype is tau phosphorylation dependant and attenuated by inhibitors of Glycogen synthase kinase-3 (GSK-3). We have thought for some time that this model is ideally suited for testing compounds that might alter axonal transport or affect signalling though GSK-3 and other kinases to tau phosphorylation. However, we had other priorities in the laboratory and this work was not pursued.

The article by Chen et al. [1] coincided with planning for an open day as part of the British Association for Advancement of Science's Science Week in March 2005 and funded by the Medical Research Council as part of a Public Engagement with Science activity. As a consequence we established a collaboration between three local schools and the Institute of Psychiatry at King's College London, and students worked alongside research workers to test a series of compounds on a larval phenotype and the neuromuscular junction (NMJ) anatomy of wild-type flies (Oregon R) and transgenic flies (human 3R tau expressed in motor neurons).

We have replicated the previous observation that tau expression significantly alters motor-dependant phenotypes and in addition that the NMJ is severely disrupted. We have tested a series of compounds that alter signalling to GSK-3, and with one exception these all alter the phenotype in the predicted direction—where inhibition of GSK-3 improves the phenotype. We also tested curcumin, which has previously been shown to alter Aβ aggregation and amyloid-dependant processes [3]. We found no effect of curcumin in our tau-dependant model. Most interestingly, we found a large and very highly significant worsening of the phenotype with taxol-treated larvae, in contrast to previous studies [4].

We, the students, believe that it was worthwhile doing the experiment as it could lead to greater understanding of Alzheimer disease, and we have found that the experience has helped some of us in our commitment to pursue a career in science and medicine.

We, the scientists, have completed a pilot experiment involving multiple data points processed rapidly and using a widely available resource—students. Twelve pairs of hands allowed us to gather in a little over a day data that would have otherwise required some weeks of research time. We are now in the process of replicating and validating the student data, concentrating on those compounds that appear most effective from the student-led experiment. We are grateful to Chen et al. [1] for directing us to this valuable human resource.
